# Unveiling multifaceted effects of *Lactobacillus* fermentation on red pitaya (*Hylocereus polyrhizus*) Pulp: An integrated in silico and in vitro-vivo study

**DOI:** 10.1016/j.fochx.2025.103057

**Published:** 2025-09-19

**Authors:** Zuman Dou, Baishun Hu, Yu Kang, Yunfen Zhu, Xiaofei Chen, Hui Niu, Shanshui Zeng, Wenyang Zhang, Qingfei Duan, Qiang Huang, Bin Zhang, Chun Chen, Xiong Fu

**Affiliations:** a Enshi Tujia and Miao Autonomous Prefecture Academy of Agricultural Sciences, Enshi 445000, China; b SCUT-Zhuhai Institute of Modern Industrial Innovation, School of Food Science and Engineering, South China University of Technology, Guangzhou 510640, China; c College of Ocean Food and Biological Engineering, Jimei University, Xiamen 361021, China; d Microbiome Medicine Center, Department of Laboratory Medicine, Zhujiang Hospital, Southern Medical University, Guangzhou 510282, China; e College of Light Chemical Industry and Materials Engineering, Shunde Polytechnic, Foshan 528333, China

**Keywords:** Red pitaya, *Lactobacillus*, Antioxidant activities, Antidiabetic activities

## Abstract

Microbial strain selection dictates the functional and sensory attributes of fermented fruit products. Here, we investigated *Lactobacillus*-mediated fermentation of red pitaya pulp, assessing its phytochemical profile, antidiabetic potential, and aroma. Our results revealed substantial interspecies variability in carbohydrate utilization and lactate synthesis (3.57–6.72 mg/mL) among probiotic strains. Fermentation-induced biotransformation of phenolic constituents significantly modulated both antioxidant capacity and carbohydrate-digestive enzyme inhibition. Docking analyses identified vanillin and 1-caffeoylquinic acid, predominantly produced by *Lactobacillus thermophilus* GRX02 and *Lactiplantibacillus plantarum* LP-28, as competitive α-amylase and α-glucosidase inhibitors. In diabetic mice, fermented juice improved glycemic control and lipid metabolism, correlating with enriched bioactive phenolics. Notably, volatile compound profiling revealed substantial enhancement of desirable aromatic notes post-fermentation. These results position lactic acid bacteria fermentation as a dual-purpose biotechnology strategy to simultaneously augment the nutraceutical value and organoleptic quality of red pitaya, offering new avenues for developing organoleptically pleasing functional beverages with antidiabetic potential.

## Highlights

• All species of *Lactobacillus* could metabolize the carbohydrates to varying degrees.
• *Lactobacillus* fermentation significantly altered the content of bioactive ingredients and antidiabetic activities.
• The aroma profiles of red pitaya pulp were also improved after fermentation with *Lactobacillus*.

## 1 Introduction

The red pitaya (*Hylocereus* spp.), a tropical fruit widely recognized as dragon fruit, is a member of the Caryophyllales order. Indigenous to Mexico and Central and South America, this species has been extensively cultivated in regions including Guatemala and the southern United States (Morais, da Silva Campelo Borges, dos Santos Lima, Martín-Belloso, & Magnani, 2019; Santos et al., 2020). Beyond its visually distinctive morphology, the fruit has attracted significant scientific and commercial interest due to its putative health-promoting properties(Song et al., 2016). Red pitaya fruit is distinguished by its high concentration of bioactive compounds, such as polyphenols, flavonoids, ascorbic acid, dietary fibers, and polysaccharides, which contribute to its purported health benefits(Kim et al., 2011). Emerging research has elucidated its multifaceted pharmacological potential, spanning hypoglycemic, antioxidant, anti-adipogenic, and immunomodulatory effects, alongside therapeutic applications in chronic disease management(Guimaraes et al., 2017; Kim et al., 2011). F Notably, dietary supplementation with red pitaya pulp has been shown to markedly attenuate insulin resistance in a diabetic rodent model(Omidizadeh et al., 2014).

The food industry frequently processes fruits and vegetables into juice, a product highly valued for its palatability and nutritional benefits(Z. M. Dou, Chen, Huang, & Fu, 2022). However, the bioactive constituents in these fruits and vegetables can be significantly influenced by the processing conditions (e.g mechanical pulping, sterilization, concentration, fermentation), such as the degradation of some polyphenol monomers, while some new monomers can also be produced (Choi, Kim, Seo, Lee, & Cho, 2012). Fermentation, a well-established food processing strategy, is widely utilized in juice production, with microbial selection playing a pivotal role in determining the final product's characteristics (Gao et al., 2019). Within the diverse array of lactic acid bacteria (LAB) employed in juice fermentation, *Lactobacillus* species are particularly prominent due to their ability to enhance functional properties and sensory profiles (de Ovalle, Cavello, Brena, Cavalitto, & González-Pombo, 2018). For example, *Lactiplantibacillus plantarum* NCU116 has been demonstrated to enrich *Momordica charantia* juice with increased phenolic compounds, flavonoids, and organic acids, thereby boosting its antioxidant capacity(Gao et al., 2019). Notably, metabolic variations among *Lactobacillus* strains can lead to divergent fermentation outcomes (Cui et al., 2019). Comparative studies reveal species-dependent effects: *Lacticaseibacillus paracasei*-fermented Ougan juice exhibits stronger antioxidant activity than that produced by *Lactiplantibacillus plantarum* (Guo, Cao, Guo, & Li, 2019), whereas no significant differences were observed between *Lacticaseibacillus casei* 01 and *Lactobacillus acidophilus* La-5 in apricot juice fermentation(Bujna, Farkas, Tran, Dam, & Nguyen, 2018). Despite these findings, the impact of different *Lactobacillus* species on red pitaya (*Hylocereus* spp.) juice fermentation remains poorly understood, highlighting a critical knowledge gap in this field.

This study systematically examined how fermentation with seven *Lactobacillus* strains influences red pitaya's phytochemical profile, antioxidant capacity, antidiabetic effects, and ability to suppress advanced glycation end-product (AGE) formation, alongside assessing its sensory characteristics. Furthermore, we aimed to establish a correlation between chemical constituents and biological activities. The findings advance our understanding of microbial fermentation in modulating the functional properties of red pitaya, offering potential applications in nutraceutical development.

## 2 Materials and methods

### 2.1 Reagents

1,1-Diphenyl-2-picrylhydrazyl (DPPH), 2,2′-azinobis-(3-ethylbenzthiazoline-6-sulphonate) (ABTS), 6-hydroxy-2,5,7,8-tetramethylchroman-2-carboxylic acid (Trolox), bovine serum albumin (BSA), methylglyoxal (MGO) and porcine pancreatic α-amylase (50 U/mg) were all acquired from Sigma-Aldrich Corp. (St. Louis, MO, USA). *Saccharomyces cerevisiae*-derived α-glucosidase (23.5 U/mg), aminoguanidine hydrochloride (AG), acarbose, along with analytical standards for organic acids, free sugar and phenolic acids, were sourced from Shanghai Yuanye Biological Technology Co., Ltd. (Shanghai, China). All solvents and additional chemicals were of either analytical grade or HPLC purity.

### 2.2 Samples preparation and fermentation

Red pitaya (*Hylocereus polyrhizus*) fruits were sourced from a commercial supplier in Guangzhou, China. After thorough rinsing with deionized water, the fruits were sectioned and mechanically homogenized using a household blender. Sterile water was added to the pulp at a 1:1 (w/w) ratio, and the pH was normalized to 6.8 via dropwise addition of 1 M NaOH, yielding fresh samples (referred to as “fresh fruit”). This suspension was subsequently heat-treated at 65 °C for 30 min to generate the pasteurized control group (“p-Fresh”) (Morais et al., 2019). Microbial contamination was rigorously excluded by aseptically coating culture plates with diluted pulp prior to fermentation experiments.

Lyophilized powders of seven *Lactobacillus* strains: *Lacticaseibacillus casei* LC122 (*L. casei* LC122), *Lacticaseibacillus paracasei* LPC48 (*L. paracasei* LPC48), *Lacticaseibacillus rhamnosus* LRH09 (*L. rhamnosus* LRH09), *Lactobacillus acidophilus* LA1063 (*L. acidophilus* LA1063), *Lactobacillus thermophilus* GRX02 (*L. thermophilus* GRX02), *Lactiplantibacillus plantarum* LP-28 (*L. plantarum* LP-28) and *Lactiplantibacillus plantarum* TWK 10 (*L. plantarum* TWK 10), were kindly provided by Synbio Tech Inc. (Taiwan, China). For inoculum preparation, each powder was reconstituted in sterile deionized water (1:50, *w*/*v*) and introduced into red pitaya pulp to yield a final concentration of ~10^9^ CFU/mL. Fermentation proceeded at 37 °C for 48 h under aseptic conditions, with samples collected at 6, 12, 24, and 48 h for pH analysis.

### 2.3 pH and organic acid determination

The pH of the fermented pulp was determined using a pH meter (PHS-3C, Leici Co., Ltd., Shanghai, China). Organic acid profiles were analyzed via high-performance liquid chromatography (HPLC) using an Agilent 1260 system (Agilent Technologies, USA) equipped with a diode array detector (DAD), following a previously reported method with minor modifications (Zeppa, Conterno, & Gerbi, 2001). Separation was achieved on a ZORBAX SB-C18 column (Agilent, 4.6 × 250 mm, 5 μm) maintained at 30 °C. The mobile phase consisted of 0.01 M KH₂PO₄ (pH 2.5, solvent A) and methanol (solvent B) in a ratio of 97:3 (*v*/v), delivered at a flow rate of 1 mL/min with an injection volume of 5 μL. Detection was performed at 212 nm.

### 2.4 Measurement of total carbohydrate and reducing sugar contents

Following a 48 h fermentation period, all fermented pulp samples were harvested and subjected to centrifugation (4000 *g*, 5 min). The resulting supernatants were subsequently analyzed for carbohydrate content. Total carbohydrate quantification was performed according to the established phenol‑sulfuric acid protocol (Dou et al., 2025; Niu et al., 2025), while reducing sugar levels were determined using the dinitrosalicylic acid (DNS) assay (Miller, 1959).

### 2.5 Analysis of free sugars by HPLC

The fermentation supernate was processed as outlined in Section 2.4, with filtration through a 0.22 μm hydrophilic membrane. Chromatographic separations were performed on an Agilent 1260 HPLC system (Santa Clara, CA, USA) fitted with a refractive index detector, utilizing a ZORBAX SB-C18 column (4.6 × 250 mm, 5 μm particle size) under conditions adapted from prior methodologies(Li et al., 2022; Morais, da Silva Campelo Borges, dos Santos Lima, Martín-Belloso, & Magnani, 2019; Zu-Man, Yu-Long, Chun-Yang, Chuang, Jia-Qin, Qiang, et al., 2024).

### 2.6 Determination of total phenolic (TPC) and flavonoid (TFC) contents

Total phenolic content (TPC) was determined spectrophotometrically using the Folin-Ciocalteu assay (Singleton & Rossi, 1965). TPC was quantified against a gallic acid standard and expressed as gallic acid equivalents per litre (mg GAE/L). Total flavonoid content (TFC) was assessed using an adapted colorimetric method (Dou et al., 2022). TFC was calculated relative to a rutin standard and reported as rutin equivalents per litre (mg RE/L).

### 2.7 Characterization and quantitation of main phenolics

Phenolic compounds were quantified using an Agilent 1260 HPLC system (Agilent Technologies, USA) with a diode array detector (DAD) (Omedi, Huang, & Zheng, 2019). Chromatographic separation was performed on a ZARBAX SB-C18 column (4.6 × 250 mm, 5 μm; Agilent) under controlled conditions (30 °C). The mobile phase comprised two components: (A) 0.1 % (*v*/v) formic acid in water and (B) acetonitrile, delivered at 0.8 mL/min with the following gradient profile: 5–10 % B (0–10 min), 10–20 % B (10–15 min), 20–38 % B (15–25 min), 38–40 % B (25–30 min), 40–100 % B (30–31 min), isocratic 100 % B (31–35 min), followed by re-equilibration (100–5 % B, 35–36 min; 5 % B, 36–50 min). Detection wavelengths were optimized for target analytes: 520 nm for anthocyanins and 280 nm for other polyphenols.

### 2.8 Aroma profiles analysis

The volatile compounds in the fermented pulp were analyzed using an Agilent 7890 A gas chromatograph coupled with a 5975 mass spectrometric detector (Agilent Technologies), following a previously described method with modifications (Gao et al., 2019). Briefly, headspace solid-phase microextraction (HS-SPME; 50/30 μm DVB/CAR/PDMS fiber, Supelco, Bellefonte, PA, USA) was employed for compound extraction at 80 °C for 60 min. Separation was achieved using a Ptx-Wax capillary column (30 m × 0.25 mm, Shimadzu, Japan) with the following temperature gradient: initial hold at 35 °C for 5 min, followed by heating at 4 °C/min to 80 °C (2 min hold), then 6 °C/min to 100 °C, and finally 10 °C/min to 250 °C (5 min hold). Helium served as the carrier gas (0.8 mL/min, constant flow). The transfer line and quadrupole temperatures were set at 280 °C and 150 °C, respectively. Tentative identification of aroma compounds was based on retention indices and mass spectral matching against the NIST11 database (National Institute of Standards and Technology, Gaithersburg, MD, USA) (Gao et al., 2019).

### 2.9 Antioxidant activities

Antioxidant capacities of fermentation supernatants were assessed via DPPH and ABTS radical scavenging assays, performed according to established protocols (Dou et al., 2022).

For DPPH radical scavenging assay, a total of 100 μL of sample (diluted to appropriate concentration) was mixed with 100 μL of methanolic DPPH solution (200 μM). Subsequently, the mixture was incubated at room temperature for 30 min in absence light and then the absorbance was recorded at 517 nm. Trolox was used as a standard, and the results were calculated as μmol Trolox equivalents per millilitre of sample (μM TE/mL, sample).

For ABTS radical scavenging assay, ABTS^+^ radical was prepared by mixing 7 mM ABTS solution and 2.45 mM K_2_S_2_O_8_ solution in equal volume, then incubated in darkness at room temperature for 12 h, the resulting solution was diluted to yield a working solution with 0.7 ± 0.02 absorbance at 734 nm. Subsequently, 30 μL of sample with appropriate concentration was mixed with 225 μL of ABTS^+^ solution. After reacting for 30 min, the absorbance was measured at 734 nm. Trolox was used as a standard, and the results were calculated as μmol Trolox equivalents per millilitre of sample (μM TE/mL, sample).

### 2.10 Antidiabetic activities in vitro

#### 2.10.1 α-Amylase and α-glucosidase inhibitory activity

α-Amylase inhibition was assessed using a modified version of an established protocol (Wang et al., 2018), with acarbose serving as the positive control. Reaction mixtures comprised 20 μL sample, 80 μL starch solution (2 g/L) and 20 μL α-amylase (10 U/mL) in 0.1 M phosphate buffer (pH 6.9), following 20-min incubation at 37 °C, reactions were halted by adding 80 μL 0.4 M HCl and 100 μL 5 mM I₂-KI solution. Absorbance at 620 nm was measured, with acarbose-equivalent inhibitory capacity expressed as mg AE/L.

Parallel assays for α-glucosidase inhibition followed reported methodologies (Dou et al., 2022; Dou, Chen, Huang and Fu, 2021a, Dou, Chen, Huang and Fu, 2021b). Reaction mixtures contained 50 μL sample, 50 μL α-glucosidase (0.5 U/mL) and 50 μL pNPG (5 mM) in 0.1 M phosphate buffer (pH 6.9). After sequential incubations (10 min enzyme pre-incubation, 10 min substrate reaction) at 37 °C. Absorbance at 405 nm was recorded, with inhibitory activities quantified as μg AE/L.

#### 2.10.2 Molecular docking analysis

Pearson's correlation analysis was conducted to evaluate the relationships between individual phenolic compounds and their inhibitory effects on α-amylase and α-glucosidase. Phenolic derivatives demonstrating significant positive correlations were subsequently subjected to molecular docking simulations to investigate their binding interactions with the target enzymes. For these simulations, the crystal structures of α-amylase (PDB ID: 1OSE) and α-glucosidase (PDB ID: 3A4A) were obtained from the RCSB Protein Data Bank (http://www.rcsb.org/) (Lin et al., 2019). The three-dimensional conformations of the phenolic ligands were generated using ChemBio3D Ultra 14.0 and optimized via MM2 energy minimization (Duan et al., 2023). Prior to docking, protein structures were preprocessed using AutoDockTools-1.5.6, involving the removal of water molecules, addition of polar hydrogens, and assignment of Gasteiger partial charges. Molecular docking was performed using AutoDock Vina (v4.2.6), the binding site interactions were visualized and analyzed using Discovery Studio 2016.

#### 2.10.3 Anti-glycation assay

A 1 mL aliquot of the sample was incubated with 3 mL of methylglyoxal (MGO; 60 mM in 0.2 M phosphate buffer, pH 7.4, containing 0.02 % NaN_3_) at 37 °C for 2 h. Following this, 3 mL of bovine serum albumin (BSA; 30 mg/mL) was introduced, and the reaction mixture was further incubated for 72 h. luorescence intensity was quantified using a fluorescent microplate reader with excitation/emission wavelengths set to 350/425 nm. Aminoguanidine hydrochloride (AG) was employed as a positive control. The inhibitory effect on advanced glycation end-product (AGE) formation was assessed as follows(Spinola, Pinto, Llorent-Martinez, Tomas, & Castilho, 2019):

$$\text{Inhibition}\;\left(\%\right)=\frac{\left({\mathrm{FI}}_{C}-{\mathrm{FI}}_{B}\right)-\left({\mathrm{FI}}_{S}-{\mathrm{FI}}_{\mathrm{BS}}\right)}{{\mathrm{FI}}_{C}-{\mathrm{FI}}_{B}}\times 100\%$$

where FI_S_ and FI_BS_ represent the fluorescence intensities of the test sample and its corresponding blank, respectively, while FI_C_ and FI_B_ denote those of the control and blank groups.

### 2.11 Antidiabetic activities in vivo

Six-week-old male db/db mice and wild-type db/m littermates were maintained in a controlled environment (24–25 °C, 55–70 % humidity) at the Animal Experimental Center of Guangdong Pharmaceutical University (Guangzhou, China). The animals had ad libitum access to water and were fed a standard diet (Ethics Approval No. SPF2017411). Following a one-week acclimatization period, db/db mice were randomly allocated into nine experimental groups (*n* = 6 per group). Each group received a daily oral gavage of 250 μL of fermented red pitaya pulp for 14 consecutive days, while the control group was administered an equivalent volume of normal saline. Body weight was monitored at two-day intervals throughout the study.

#### 2.11.1 Measurement of fasting plasma glucose and insulin

Following a 12-day gavage period, mice were subjected to a 12-h fasting protocol before blood collection for the assessment of fasting blood glucose and insulin levels. The homeostasis model assessment of insulin resistance (HOMA-IR) serves as a key indicator of glucose-insulin dynamics across tissues, with elevated values correlating with increased diabetic severity. In this study, HOMA-IR was computed using the established equation: HOMA-IR = fasting plasma glucose * fasting insulin /22.5.

#### 2.11.2 Measurement of oral glucose tolerance

Prior to the conclusion of the experiment, mice in each treatment group underwent a 12-h fasting period, followed by oral administration of a glucose solution (2 g/kg). Blood glucose levels were subsequently assessed at 0, 30, 60, 90, and 120 min post-administration.

#### 2.11.3 Measurement of serum biochemical parameters

Upon completion of the experimental protocol, mice were humanely euthanized via gradual-fill carbon dioxide anesthesia in compliance with EU Directive 2010/63/EU. Serum biomarkers, including insulin, high-density lipoprotein cholesterol, low-density lipoprotein cholesterol, total cholesterol, and triglycerides, were quantified using commercially available assay kits (Dou et al., 2023).

### 2.12 Statistical analysis

Statistical analyses were performed using IBM SPSS Statistics (v24.0; IBM Corp., USA). Between-group comparisons were evaluated through one-way analysis of variance (ANOVA), with post hoc testing conducted using Duncan and LSD methods. Results are expressed as mean ± standard deviation (SD) derived from triplicate experimental determinations.

## 3 Results and discussion

### 3.1 pH and organic acid changes

**Fig. 1:**
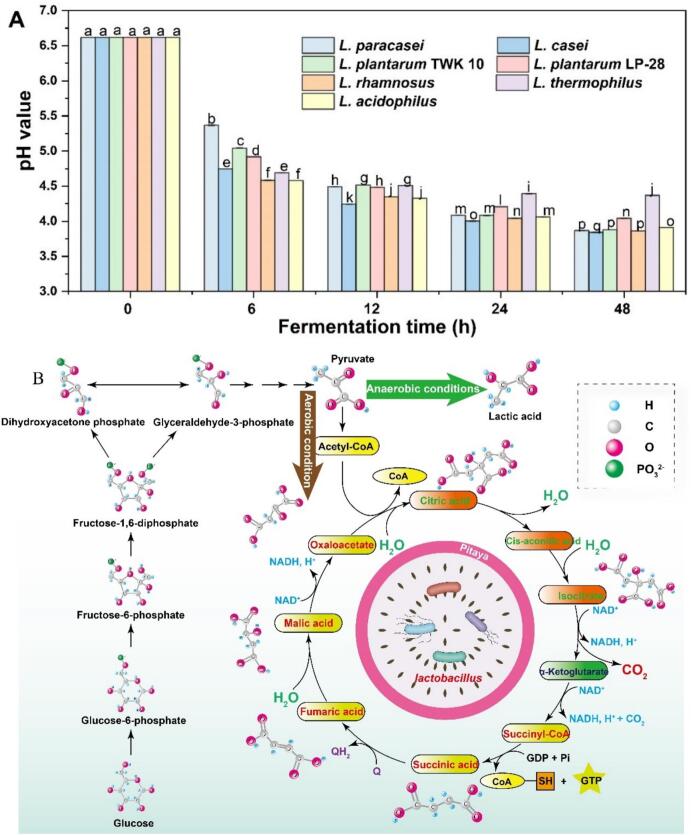
(A) pH changes of red pitaya pulp during the fermentation with various strains of *Lactobacillus*, different lowercase letters indicate significant differences among the different groups at the *P* < 0.05 level; (B) The mechanism of pH reduction of red pitaya pulp (The figure was drawn according to the literature: *Trends in biotechnology*, 2003, 21(6): 269–274). (For interpretation of the references to colour in this figure legend, the reader is referred to the web version of this article.)

Compared to fresh juice (pH 6.82), the pasteurized sample exhibited a notable decline in pH (6.62), likely due to increased organic acid concentrations (Table S1). In red pitaya pulp, carbohydrates undergo glycolysis to generate pyruvate, which may subsequently be metabolized by *Lactobacillus* into lactic acid via fermentation or enter the tricarboxylic acid cycle under anaerobic conditions, thereby contributing to acidification (Welman & Maddox, 2003). The underlying biochemical pathway is detailed in Fig. 1B. Furthermore, pH dynamics, which serve as a key indicator of fermentation progression, were systematically monitored and are presented in Fig. 1A.

At the onset of fermentation (0 h), all experimental groups displayed an initial pH of 6.62. Within the first 6 h, a sharp decline in pH was observed, after which the reduction proceeded more gradually (Fig. 1A)-a trend consistent with prior reports on *Lactiplantibacillus plantarum*-mediated fermentation of liquorice root extract (Mousavi & Mousavi, 2019). This rapid acidification likely stems from the accumulation of acidic metabolites coupled with diminished buffering capacity due to exponential bacterial growth. By the end of the 48-h fermentation period, significant inter-strain differences emerged. The L. *thermophilus* GRX02 group maintained the highest pH, whereas *L. plantarum* LP-28 yielded a moderately lower value (pH = 4.05). In contrast, no statistically significant variation (*p* > 0.05) was detected among the *L. paracasei* LPC48, *L. casei* LC122, *L. plantarum* TWK10, and *L. rhamnosus* LRH09 groups, all of which reached a comparable final pH (3.87). These findings underscore the strain-specific influence of *Lactobacillus* spp. on the fermentation dynamics of red pitaya pulp.

Table S1 delineates the dynamic shifts in organic acid profiles within red pitaya pulp under varying processing conditions. In fresh fruit, malic acid predominated at 1.69 ± 0.05 mg/mL but underwent a significant reduction (*p* < 0.05) to 1.58 ± 0.02 mg/mL post-pasteurization, likely due to thermal degradation into secondary metabolites, including tartaric, acetic, citric, and succinic acids (Chu & Clydesdale, 1976). Notably, fermentation with diverse *Lactobacillus* strains universally depleted malic acid, concomitant with its bioconversion into lactic acid-a metabolic trend consistent with prior observations in *Momordica charantia* juice fermented by L. *plantarum* NCU 116 (Gao et al., 2019). Lactic acid yields varied substantially across strains (*L. paracasei* LPC48: 6.24 ± 0.11 mg/mL; *L. casei* LC122: 5.41 ± 0.08 mg/mL; *L. plantarum* TWK10: 5.75 ± 0.03 mg/mL; *L. plantarum* LP-28: 4.72 ± 0.04 mg/mL; *L. rhamnosus* LRH09: 6.72 ± 0.03 mg/mL; *L. thermophilus* GRX02: 3.57 ± 0.05 mg/mL; *L. acidophilus* LA1063: 4.95 ± 0.03 mg/mL). These values represent an 8.71–18.67-fold elevation over fresh fruit (0.41 ± 0.01 mg/mL) and a 9.12–18.67-fold increase relative to pasteurized pulp (0.36 ± 0.02 mg/mL), underscoring the metabolic efficiency of lactic acid bacteria in substrate utilization.

Comparative analysis revealed a modest elevation in acetic acid content in fermented samples relative to fresh fruit (Table S1). This metabolic shift is physiologically relevant, as acetic acid has been demonstrated to improve glucose tolerance and stimulate insulin secretion in rodent models of diet-induced metabolic dysfunction (L. Wang, Li, Huang, & Fu, 2020). These findings position *Lactobacillus*-mediated fermentation of red pitaya pulp as a viable strategy for developing novel functional foods with potential antidiabetic applications. Furthermore, we observed a concomitant reduction in malic acid concentration following fermentation, consistent with prior reports(Mousavi & Mousavi, 2019). This phenomenon likely reflects the enzymatic conversion of malic acid to lactic acid via malolactic activity during microbial processing (Gao et al., 2019).

### 3.2 Metabolism of carbohydrates

**Fig. 2:**
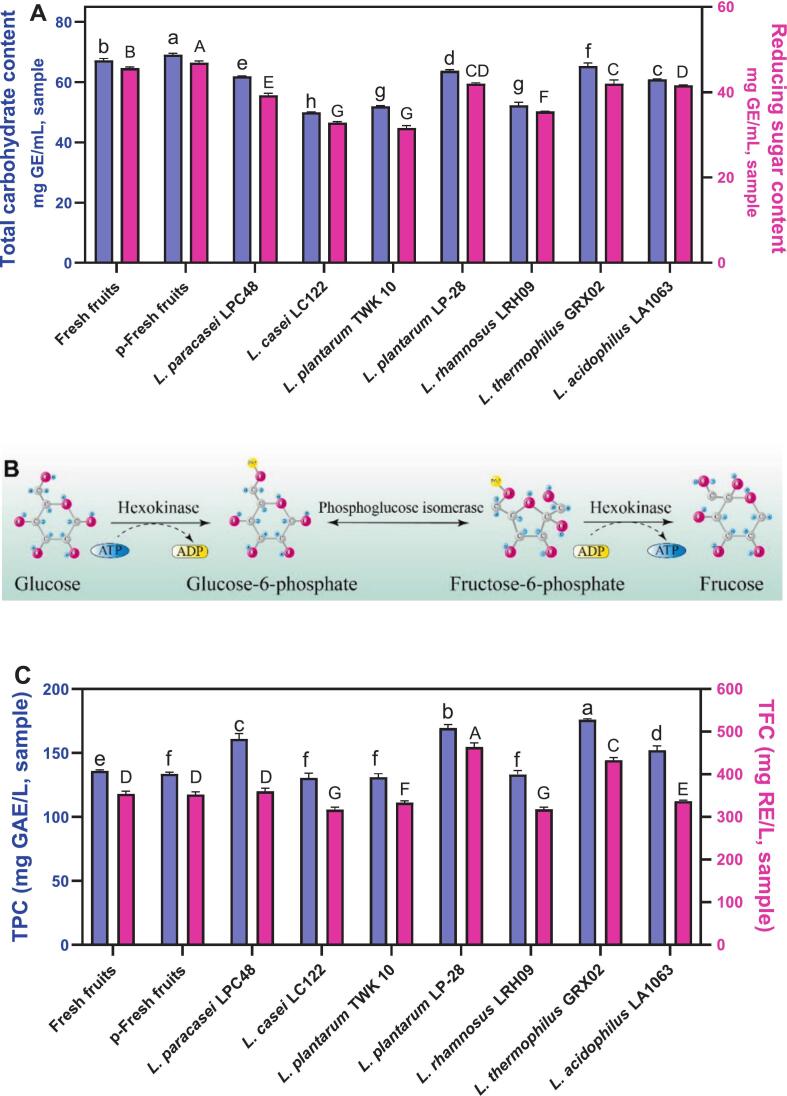
The changes in total carbohydrates and reducing sugar contents, different lowercase letters indicate significant differences in total sugar content at the *P* < 0.05 level among different groups, while different capital letters represent significant differences in reducing sugar content at the *P* < 0.05 level among different groups (A); the mechanism of glucose converts into fructose (B); total phenolic contents (TPC) and total flavonoid contents (TFC) in red pitaya pulp after fermentation, different lowercase letters indicate significant differences in TPC content at the *P* < 0.05 level among different groups, while different capital letters represent significant differences in TFC content at the *P* < 0.05 level among different groups (C). (For interpretation of the references to colour in this figure legend, the reader is referred to the web version of this article.)

Carbohydrates are metabolized to pyruvate, which serves as a precursor for lactic acid and other organic acids (Welman & Maddox, 2003). Consequently, carbohydrate utilization reflects the dynamic changes in organic acid profiles and medium acidification. As depicted in Fig. 2A, the initial total carbohydrate content in fresh fruit (67.28 ± 0.61 mg GE/mL) increased slightly in p-fresh fruit (69.13 ± 0.48 mg GE/mL), likely due to heat-induced liberation of small-molecular-weight sugars from pitaya fiber(Gao et al., 2019). However, fermentation led to a significant (*p* < 0.05) reduction in total carbohydrate content. A similar trend was observed for reducing sugars (Fig. 2A), with varying degrees of depletion across fermentation groups, consistent with differences in pH and organic acid accumulation(Mousavi & Mousavi, 2019). These observations indicate that *Lactobacillus* preferentially metabolizes reducing sugars from the available carbohydrate pool.

High-performance liquid chromatography (HPLC) was employed to investigate the fluctuations in free sugar composition throughout the fermentation process of red pitaya pulp. As summarized in Table S2, the predominant sugars detected were fructose, glucose, and maltose, with glucose constituting the most abundant fraction. Fructose and maltose were present at nearly equivalent concentrations, aligning with previously reported data(Morais et al., 2019). A marginal elevation in glucose and maltose levels was observed following pasteurization, contributing to the higher total carbohydrate and reducing sugar content in pasteurized fresh fruit (p-fresh fruit) compared to untreated samples. Post-fermentation, all three sugars exhibited varying degrees of depletion, confirming their simultaneous utilization by *Lactobacillus* strains. Notably, glucose consumption exceeded that of fructose and maltose, consistent with its well-documented role as the preferred carbon source for microbial metabolism (Lu, Fleming, & McFeeters, 2001). Consequently, fructose and maltose were metabolized secondarily in the presence of glucose (Brückner & Titgemeyer, 2002). Among the tested strains, *L. plantarum* LP-28 and L. *thermophilus* GRX02 demonstrated relatively low glucose utilization rates (19.64 % and 20.96 %, respectively), correlating with the observed trends in total carbohydrate and reducing sugar content, as well as concomitant pH and organic acid shifts (Mousavi & Mousavi, 2019). Intriguingly, fructose levels increased in the L. *plantarum* LP-28, *L. thermophilus* GRX02, and L. *acidophilus* LA1063 fermentation groups, likely due to the enzymatic conversion of glucose to fructose during fermentation (Fig. 2B) (Welman & Maddox, 2003).

### 3.3 Changes of phenolics and flavonoids

Red pitaya is a rich source of bioactive phenolic compounds, including cyanidin-3-glucoside and gallic acid, which are known to confer health-promoting properties in food applications (Gao et al., 2019; Morais et al., 2019). To assess the impact of processing on these phytochemicals, we quantified the total phenolic (TPC) and flavonoid (TFC) contents (Fig. 2C). While pasteurization induced only a marginal reduction in TPC (from 136.0 ± 0.67 to 133.67 ± 1.21 mg GAE/L), consistent with the relative thermal stability of phenolic constituents, fermentation elicited more pronounced changes. Notably, treatment with *L*. *paracasei* LPC48, *L. plantarum* LP-28, *L. thermophilus* GRX02, and *L. acidophilus* LA1063 significantly enhanced TPC by 1.18- to 1.30-fold relative to fresh fruit, likely due to microbial enzymatic modification (e.g., decarboxylase, tannase, and reductase activity) facilitating the liberation or bioconversion of phenolic moieties(Gao et al., 2019). In contrast, fermentation with *L. casei* LC122, *L. plantarum* TWK10, and *L. rhamnosus* LRH09 yielded negligible TPC alterations, highlighting strain-dependent metabolic specificity(Nie et al., 2017). A parallel trend was observed for TFC, with *L. thermophilus* GRX02 and *L. plantarum* LP-28 exhibiting the most substantial increases (1.23- and 1.31-fold, respectively) over baseline levels (354.16 ± 6.05 mg RE/L).

HPLC analysis revealed the presence of 11 phenolic compounds in fresh fruit, with 2,4,6-trihydroxybenzoic acid, catechol, and quercetin-3-O-glucoside constituting the major components (Table S3). Pasteurization led to a marked reduction in phenolic content, attributable to thermal degradation, consistent with the observed decline in TPC and TFC content compared to fresh fruit. Intriguingly, fermentation with *Lactobacillus* strains induced differential increases in phenolic concentrations, though the overall diversity of detected compounds remained stable. This highlights species-specific metabolic transformations of phenolics by *Lactobacillus*(Gao et al., 2019). Notably, 2,4,6-trihydroxybenzoic acid exhibited a significant (*p* < 0.05) post-fermentation increase, likely due to microbial hydrolysis of glycosylated or complex phenolic precursors (Mousavi & Mousavi, 2019). Similarly, catechol accumulation correlated with microbial decarboxylation of protocatechuic acid (Rodríguez, Landete, Rivas, & Muñoz, 2008), which itself showed a modest rise, possibly via demethylation and dehydrogenation of caffeic acid and vanillin under acidic conditions (Fig. S1) (Zhang et al., 2019). Further microbial modifications included the conversion of syringic acid to vanillin via demethylation and dihydroxylation, alongside tannase-mediated hydrolysis of gallotannins to yield gallic acid (Curiel et al., 2015). In contrast, cyanidin-3-O-glucoside and chlorogenic acid remained stable across fermented samples. Strikingly, quercetin-3-O-glucoside was undetectable in *L. plantarum* TWK 10 and LP-28 fermentations, suggesting microbial degradation or biotransformation (Zhang et al., 2019).

### 3.4 Aroma component analysis

Aroma, a critical determinant of sensory quality in fruit-derived products, plays a pivotal role in shaping consumer preferences. Extensive studies have established that lactic acid bacteria (LAB) fermentation can significantly modulate the volatile composition of fruits and vegetables(Gao et al., 2019). For instance, Obenland et al. characterized the volatile profiles of various pitaya cultivars, revealing 34 distinct compounds, predominantly aldehydes, hydrocarbons, alcohols, ketones, esters, and furans(Obenland et al., 2016). In the present study, we detected 107 volatile metabolites across fresh pitaya, pasteurized fresh fruit, and LAB-fermented products (Table S4), encompassing 1 phenolic compound, 14 alcohols, 7 aldehydes, 15 ketones, 41 hydrocarbons, 21 carboxylic acids, 7 esters, and 1 ether. Strikingly, fermentation with *Lactobacillus* spp. markedly elevated phenol levels, which constituted approximately 60 % of the total relative peak area, underscoring its transformative impact on aroma biosynthesis.

**Fig. 3:**
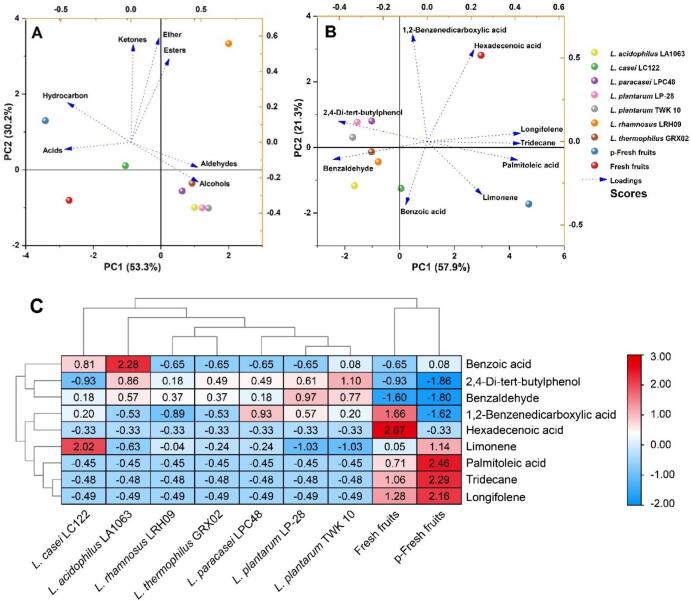
Principal component analysis (PCA) of different classification of aroma compounds (A) and score plot of aroma compounds (B) in red pitaya pulp, heatmap visualization and hierarchical clustering analysis of aroma compounds in red pitaya pulp fermented with *Lactobacillus* (C). (For interpretation of the references to colour in this figure legend, the reader is referred to the web version of this article.)

Principal component analysis (PCA) was employed to evaluate the classification of aroma compounds, revealing clear differentiation among sample groups (Fig. 3A). The first two principal components, PC1 (50.6 %) and PC2 (30.3 %), explained 80.9 % of the total variance, with PC1 demonstrating a pronounced separation between fresh fruit and fermented samples, indicative of substantial fermentation-induced modifications in aroma profiles. In contrast, PC2 distinguished fresh fruit from pasteurized fresh fruit (p-fresh fruit), the latter being characterized by elevated levels of acids and hydrocarbons, suggesting that pasteurization significantly influences the volatile composition of pitaya juice. Notably, fermentation with *L. paracasei* LPC48, *L. acidophilus* LA1063, *L. thermophilus* GRX02, *L. plantarum* LP-28, and *L. plantarum* TWK10 resulted in closely clustered PCA distributions, with aldehydes and phenols dominating their aroma profiles. In contrast, fermentation by *L. casei* LC122 and *L. rhamnosus* LRH09 yielded distinct separations along PC1 and PC2, respectively, with their metabolic outputs primarily comprising phenols, hydrocarbons, ketones, ethers, esters, and alcohols. These findings highlight the species-specific metabolic contributions of Lactobacillus strains to aroma compound formation, emphasizing the intricate relationship between microbial activity and flavor development.

Among the detected aroma compounds, nine major constituents (relative integral area ≥ 3 %) were selected to assess the impact of *Lactobacillus* fermentation on aromatic profiles. As shown in Fig. 3B, significant differences were observed in the distribution of volatile compounds across fresh fruit, pasteurized fruit (p-fresh fruit), and fermented samples. The fresh fruit was predominantly characterized by 1,2-benzenedicarboxylic acid and hexadecenoic acid (carboxylic acids), whereas the p-fresh fruit exhibited a distinct profile dominated by limonene, palmitoleic acid, tridecane, and longifolene (hydrocarbons and acids). Following fermentation, the aromatic composition shifted markedly, with 2,4-di-tert-butylphenol, benzaldehyde, and benzoic acid (phenol, aldehyde, and acid, respectively) emerging as the predominant compounds. These findings align with principal component analysis (PCA) of aroma compound classification, confirming that both pasteurization and fermentation significantly alter the volatile profile of red pitaya.

Heatmap analysis (Fig. 3C) demonstrated that phenols represented the most abundant volatile class in fermented samples, with 2,4-di-tert-butylphenol—a compound characterized by a pronounced floral aroma—constituting nearly 60 % of the total volatile composition (Wang et al., 2019). Aldehydes were the second most dominant group, wherein benzaldehyde accounted for approximately 10 % of key aroma-active compounds. This increase in benzaldehyde likely stems from the enzymatic conversion of phenylalanine via aminotransferase activity (Groot & de Bont, 1998), a compound known to confer almond and caramelized sugar nuances(Gao et al., 2019). Notably, palmitoleic acid levels exhibited a marked decline post-fermentation but rebounded significantly following pasteurization, a shift correlated with lard-like olfactory attributes. Additionally, samples fermented with *L. casei* LC122 and subsequently pasteurized displayed elevated limonene concentrations, imparting distinct citrus and mint tones(Zheng, Zhang, Quan, Zheng, & Xi, 2016). Collectively, these findings indicate that *Lactobacillus*-mediated fermentation enhances the aromatic complexity of red pitaya pulp, refining its overall sensory profile.

### 3.5 Antioxidant activity analysis

**Fig. 4:**
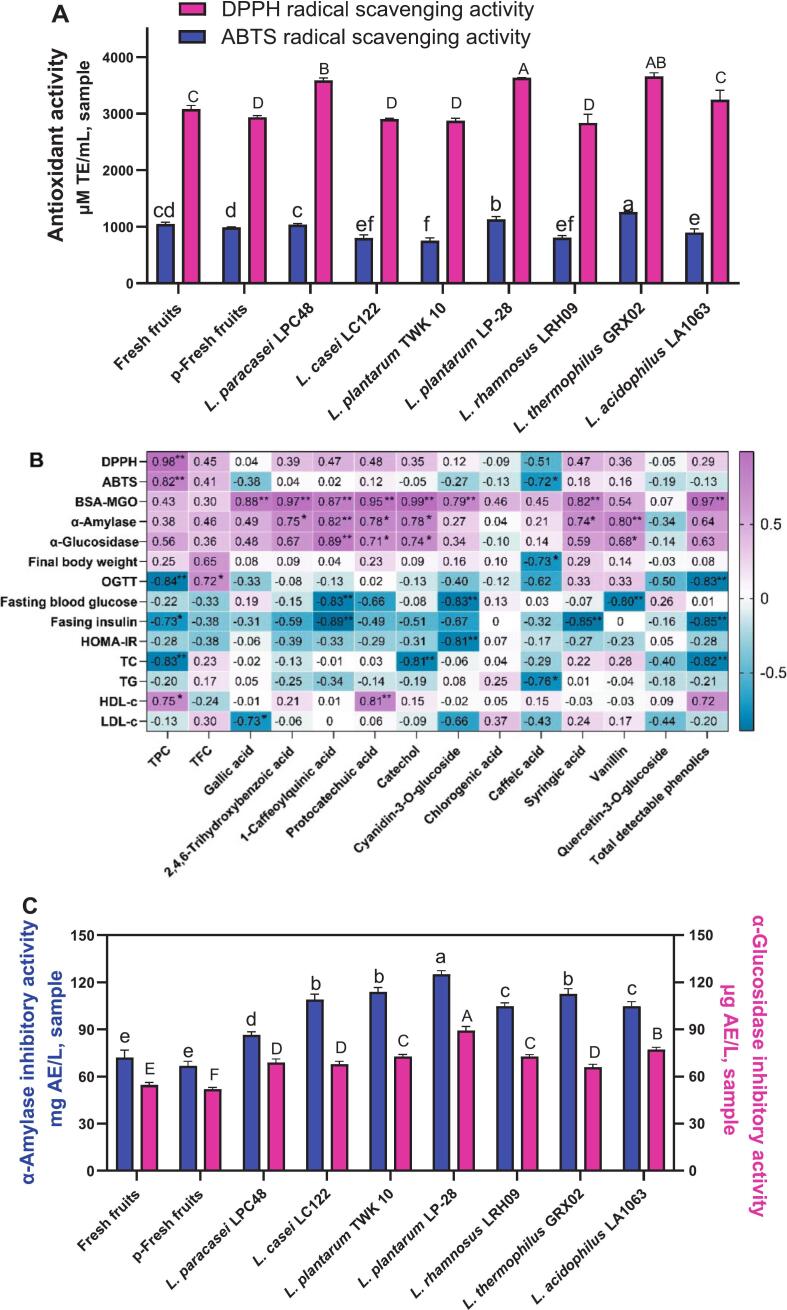
The changes in antioxidant activities: DPPH radical scavenging activity and ABTS radical scavenging activity, different lowercase letters indicate significant differences in ABTS radical scavenging activity at the *P* < 0.05 level among different groups, while different capital letters represent significant differences in DPPH radical scavenging activity at the *P* < 0.05 level among different groups (A). Pearson's correlation coefficient analysis between released bioactive compounds and bio-activities from red pitaya pulp (B). * Correlation was significant at the 0.05 level (two-tailed); ** Correlation was significant at the 0.01 level (two-tailed). In vitro antidiabetic activities analysis: Inhibitory effects on α-amylase and α-glucosidase, different lowercase letters indicate significant differences in α-amylase inhibitory activity at the *P* < 0.05 level among different groups, while different capital letters represent significant differences in α-glucosidase inhibitory activity at the *P* < 0.05 level among different groups (C). (For interpretation of the references to colour in this figure legend, the reader is referred to the web version of this article.)

To evaluate the impact of pasteurization and *Lactobacillus* fermentation on the antioxidant properties of red pitaya pulp, two established radical scavenging assays (DPPH and ABTS) were employed. As illustrated in Fig. 4A, fermentation with *L. plantarum* LP-28 and *L. thermophilus* GRX02 yielded the highest DPPH and ABTS radical scavenging activity, surpassing that of *L. paracasei* LPC48. In contrast, a marked decline (*p* < 0.05) in antioxidant capacity was observed in samples fermented with *L. casei* LC122, *L. plantarum* TWK10, and *L. rhamnosus* LRH09 compared to fresh fruit. Pasteurization alone resulted in a modest reduction in radical scavenging activity. Pearson correlation analysis revealed a robust positive relationship between total phenolic content (TPC) and antioxidant activity (Fig. 4B). Specifically, TPC exhibited a strong correlation with DPPH (*r* = 0.976, *p* < 0.01) and ABTS (*r* = 0.823, p < 0.05) scavenging capacities, consistent with previous findings(Gao et al., 2019). These data suggest that elevated TPC levels contribute to enhanced antioxidant effects. However, no significant association (*p* > 0.05) was detected between individual phenolic compounds and antioxidant activity.

### 3.6 Antidiabetic activity analysis in vitro

#### 3.6.1 Inhibition of α-amylase and α-glucosidase

The hydrolysis of dietary carbohydrates into glucose is primarily mediated by the enzymatic activity of α-amylase and α-glucosidase. Pharmacological inhibition of these enzymes represents a well-established therapeutic strategy for diabetes management, as it attenuates postprandial hyperglycemia by delaying intestinal glucose absorption (Dou et al., 2022). In this study, all experimental groups demonstrated significant enhancements in α-amylase and α-glucosidase inhibitory activity (p < 0.05). Among these, fermentation with *L. plantarum* LP-28 yielded the most pronounced effects, exhibiting 1.73- and 1.63-fold greater inhibition of α-amylase and α-glucosidase, respectively, compared to fresh fruit (Fig. 4C). These results highlight the considerable potential of microbial fermentation in augmenting the antidiabetic properties of red pitaya. Conversely, pasteurization was found to diminish hypoglycemic activity, likely due to thermal degradation of bioactive constituents, including phenolic compounds(Gao et al., 2019). This thermal instability may account for the reduced inhibitory capacity against carbohydrate-digesting enzymes. Notably, while total phenolic (TPC) and flavonoid (TFC) content showed weak correlations with enzyme inhibition (Fig. 4B), specific metabolites exhibited strong associations. In particular, 1-caffeoylquinic acid demonstrated significant positive correlations with both α-amylase (*r* = 0.823, p < 0.01) and α-glucosidase (*r* = 0.893, p < 0.01) inhibition, while vanillin was strongly correlated with α-amylase suppression (*r* = 0.798, p < 0.01). Collectively, these findings suggest that *Lactobacillus*-mediated fermentation plays a critical role in modulating the inhibitory effects of red pitaya pulp on carbohydrate-digesting enzymes. The process appears to facilitate the biosynthesis or liberation of bioactive metabolites with substantial antidiabetic efficacy (Spínola, Llorent-Martínez, & Castilho, 2019).

#### 3.6.2 Molecular docking analysis

To elucidate the inhibitory mechanisms of 1-caffeoylquinic acid and vanillin against α-amylase and α-glucosidase, molecular docking simulations were performed using the AutoDock method. As illustrated in Fig. S2, both compounds were found to occupy the hydrophobic cavity of the target enzymes, with their interactions primarily mediated by conventional hydrogen bonds, carbon‑hydrogen bonds, π-alkyl stacking, and van der Waals forces (Table S5). For α-amylase, the dominant conformations of 1-caffeoylquinic acid and vanillin exhibited binding energies of −5.06 and −4.35 kcal/mol, respectively. Given that lower binding energy correlates with higher ligand affinity (Lin et al., 2019), the stronger inhibitory effect of 1-caffeoylquinic acid (r = 0.823) compared to vanillin (r = 0.798) may be attributed to its more favorable binding thermodynamics. In the case of α-glucosidase, 1-caffeoylquinic acid demonstrated a binding energy of −4.47 kcal/mol, interacting with 14 amino acid residues. Key interactions included a hydrogen bond with ASP242 and van der Waals contacts with THR310, ASP307, SER311, PHE314, LEU313, LYS156, SER157, SER241, and LEU177. Collectively, these findings suggest that the inhibitory effects of phenolic compounds arise from their stable binding within the enzymes' active sites, predominantly via hydrogen bonding and van der Waals interactions. Such binding likely obstructs substrate access, thereby reducing enzymatic activity.

#### 3.6.3 Inhibition on AGEs formation

**Fig. 5:**
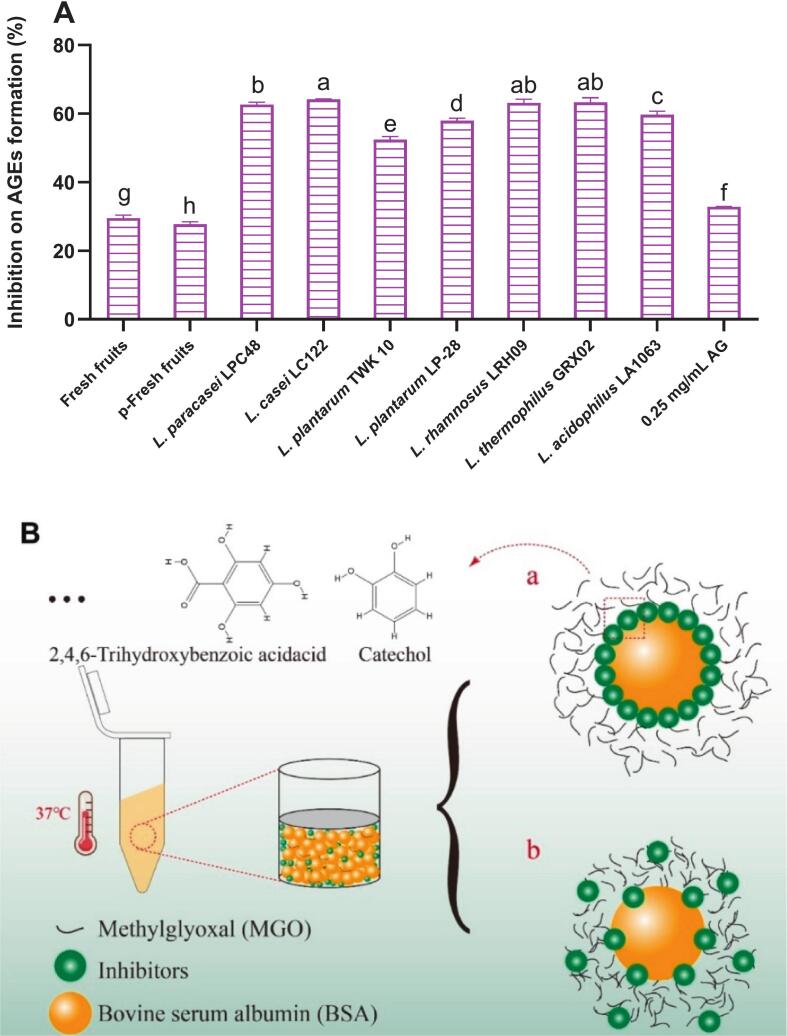
Percentage inhibition on AGEs formation of fresh fruit and fermented products, different lowercase letters indicate significant differences in AGEs inhibitory activity at the *P* < 0.05 level among different groups (A), the deduced mechanism of inhibition on AGEs formation based on BSA-MGO model (B).

Chronic hyperglycemia and associated oxidative stress in diabetic individuals promote the pathogenesis of diabetic complications—including nephropathy, cardiovascular disease, cataracts, and accelerated aging—via the non-enzymatic generation of advanced glycation end-products (AGEs) (Zhang et al., 2018). AGE formation mediates protein cross-linking and receptor interactions, triggering reactive oxygen species (ROS) overproduction and inflammatory cascades through the activation of pro-inflammatory mediators(Spinola et al., 2019). Consequently, suppressing protein glycation has emerged as a viable therapeutic approach to delay or attenuate diabetes-associated pathologies. As depicted in Fig. 5A, pasteurization diminished the antiglycation capacity of red pitaya compared to its fresh counterpart. In contrast, fermentation with *Lactobacillus* significantly enhanced AGE inhibition in the pulp, exceeding even the efficacy of the reference compound aminoguanidine (AG, 0.25 mg/mL). These results imply that fermented red pitaya may serve as a potent functional food for mitigating diabetic complications. Notably, specific phenolic compounds—rather than total phenolic content (TPC)—exhibited strong correlations with antiglycation activity (Fig. 4B). Catechol (*r* = 0.989, *p* < 0.01), 2,4,6-trihydroxybenzoic acid (*r* = 0.970, p < 0.01), and protocatechuic acid (*r* = 0.949, p < 0.01) were particularly influential, likely due to their high abundance and catechol moieties. This finding aligns with prior reports that catechol-containing phenolics demonstrate superior antiglycation properties (Nagai, Shirakawa, Ohno, Moroishi, & Nagai, 2014). Mechanistically, these inhibitors appear to function via two pathways (Fig. 5B): (i) competitive binding with methylglyoxal (MGO) for bovine serum albumin (BSA) sites (Fig. 5B-a), and (ii) formation of a stable phenolic-BSA-MGO ternary complex (Fig. 5B-b).

### 3.7 Antidiabetic activity analysis in vivo

**Fig. 6:**
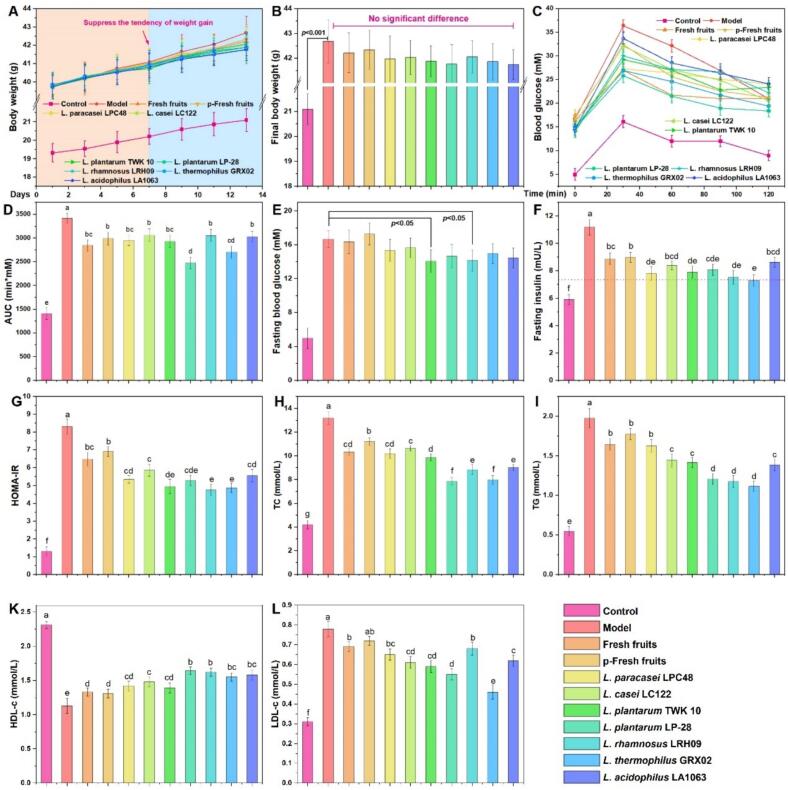
Changes in glucose and lipid metabolism in db/db mice after intervention with fresh fruit and fermented products. (A) Changes in body weight, (B) Final body weight, (C) the curves of OGTT, (D) The area under the OGTT curve, (E) Fasting blood glucose, (F) Fasting insulin, (G) HOMA-IR, (H) The content of TC, (I) The content of TG, (J) High-density lipoprotein cholesterol content, (K) Low-density lipoprotein cholesterol content. Different lowercase letters indicate significant differences at the *P* < 0.05 level among different groups.

To assess the in vivo antidiabetic potential of fermented red pitaya pulp, we employed the db/db murine model of type 2 diabetes. Initial observations revealed a transient attenuation in body weight gain following a 7-day intervention (Fig. 6A), though no significant intergroup differences were detected by the end of the 14-day treatment period (Fig. 6B).

Comparative analysis demonstrated that fermentation with select *Lactobacillus* strains: *L. plantarum* TWK 10, *L. plantarum* LP-28, *L. rhamnosus* LRH09, and L. *thermophilus* GRX02, yielded superior improvements in metabolic parameters relative to unfermented fruit. Specifically, these preparations enhanced oral glucose tolerance, reduced fasting blood glucose and insulin levels, and ameliorated insulin resistance (Fig. 6C–G). Furthermore, fermented pulp significantly modulated lipid homeostasis, as evidenced by decreased serum concentrations of total cholesterol (TC), triglycerides (TG), and low-density lipoprotein cholesterol (LDL-C), alongside elevated high-density lipoprotein cholesterol (HDL-C) (Fig. 6H–L). Pearson correlation analysis (Fig. 4B) identified robust inverse associations between glucose metabolism markers and specific phytochemicals: total phenols (vs. OGTT, *r* = −0.840), 1-caffeoylquinic acid (vs. fasting glucose, *r* = −0.835; vs. fasting insulin, *r* = −0.887), cyanidin-3-O-glucoside (vs. fasting glucose, *r* = −0.830), syringic acid (vs. fasting insulin, *r* = −0.850), and vanillin (vs. fasting glucose, *r* = −0.800). Lipid metabolism correlated strongly with total phenols (vs. TC, *r* = −0.828), protocatechuic acid (vs. HDL-C, *r* = 0.807), and catechol (vs. TC, *r* = −0.807). Given the hallmark dyslipidemia and hyperglycemia in diabetes (Wang et al., 2020; Wang et al., 2023), these findings suggest that phenolic derivatives, particularly 1-caffeoylquinic acid, cyanidin-3-O-glucoside, syringic acid, and vanillin, serve as primary hypoglycemic agents. The positive association between protocatechuic acid and HDL-C aligns with HDL's role in reverse cholesterol transport (Zu-Man et al., 2024), implicating this compound as a key modulator of lipid metabolism. Notably, *L. plantarum* LP-28 and *L. thermophilus* GRX02 emerged as particularly efficacious strains, consistent with in vitro assays. These results highlight the capacity of microbial fermentation to augment the bioactivity of red pitaya through targeted enrichment of phenolic constituents (Wang et al., 2021).

## 4 Conclusions

In this study, we present the first investigation into the fermentation of red pitaya pulp using distinct *Lactobacillus* strains. Our findings demonstrate that all tested species efficiently metabolized available carbohydrates, resulting in a marked reduction in pH and a concomitant rise in lactic acid concentration. Notably, fermentation induced significant species-dependent modifications in phenolic composition, alongside enhanced bioactivity—including elevated antioxidant capacity, antidiabetic effects (validated both in vitro and in vivo), and suppression of advanced glycation end-product (AGE) formation. Furthermore, *Lactobacillus* fermentation markedly improved the aromatic profile of the pulp. Among the evaluated strains, *L. thermophilus* GRX02 and *L. plantarum* LP-28 exhibited particularly promising potential for augmenting the functional properties of red pitaya juice, positioning them as viable candidates for developing palatable antidiabetic beverages. However, the pronounced acidity post-fermentation may compromise sensory acceptability, necessitating further optimization to refine flavor profiles without compromising bioactivity, for example, by increasing the sweetness of the juice and combining it with honey and maple syrup to create new beverages.

## CRediT authorship contribution statement

**Zuman Dou:** Writing – original draft, Supervision, Software, Resources, Methodology, Investigation. **Baishun Hu:** Writing – original draft, Methodology, Investigation. **Yu Kang:** Investigation, Formal analysis, Data curation. **Yunfen Zhu:** Software, Resources. **Xiaofei Chen:** Methodology, Investigation. **Hui Niu:** Software, Investigation. **Shanshui Zeng:** Software, Investigation. **Wenyang Zhang:** Formal analysis, Data curation. **Qingfei Duan:** Software, Formal analysis. **Qiang Huang:** Methodology, Investigation, Data curation. **Bin Zhang:** Supervision, Software, Methodology. **Chun Chen:** Methodology, Funding acquisition. **Xiong Fu:** Writing – review & editing, Funding acquisition, Conceptualization.

## Declaration of competing interest

The authors declare that they have no known competing financial interests or personal relationships that could have appeared to influence the work reported in this paper.

## Data Availability

No data was used for the research described in the article.

## Appendix A Supplementary data

Supplementary material Image 1
