# Supplementary material for: Unveiling multifaceted effects of Lactobacillus fermentation on red pitaya (Hylocereus polyrhizus) Pulp: An integrated in silico and in vitro-vivo study
Source: Food Chem X. 2025 Sep 19;31:103057. doi: 10.1016/j.fochx.2025.103057 (PMC12509195; doi:10.1016/j.fochx.2025.103057)
Supplement: Supplementary file 1 — Supplementary material [file mmc1.docx]

**Unveiling multifaceted effects of *Lactobacillus* fermentation on red pitaya (*Hylocereus polyrhizus*) Pulp: An integrated in silico and in vitro-vivo study**

Zuman Dou ^a, b, c*^, Baishun Hu ^a^, Yu Kang ^a^, Yunfen Zhu ^a^, Xiaofei Chen ^a^, Hui Niu ^b^, Shanshui Zeng ^d^, Wenyang Zhang ^e^, Qingfei Duan ^b^, Qiang Huang ^b^, Bin Zhang ^b^, Chun Chen ^b*^, Xiong Fu ^b*^

^a^ Enshi Tujia and Miao Autonomous Prefecture Academy of Agricultural Sciences, Enshi 445000, China

^b^ SCUT-Zhuhai Institute of Modern Industrial Innovation, School of Food Science and Engineering, South China University of Technology, Guangzhou 510640, China

^c^ College of Ocean Food and Biological Engineering, Jimei University, Xiamen 361021, China

^d^ Microbiome Medicine Center, Department of Laboratory Medicine, Zhujiang Hospital, Southern Medical University, Guangzhou 510282, China

^e^ College of Light Chemical industry and Materials Engineering, Shunde Polytechnic, Foshan 528333, China

*Corresponding author:

Zuman Dou: [dzman320@163.com](mailto:dzman320@163.com); Chun Chen: [chenc@scut.edu.cn](mailto:chenc@scut.edu.cn); Xiong Fu: [lfxfu@scut.edu.cn](mailto:lfxfu@scut.edu.cn)

**Fig. S1.** Proposed catabolic pathway of phenolics released from red pitaya pulp with fermentation.


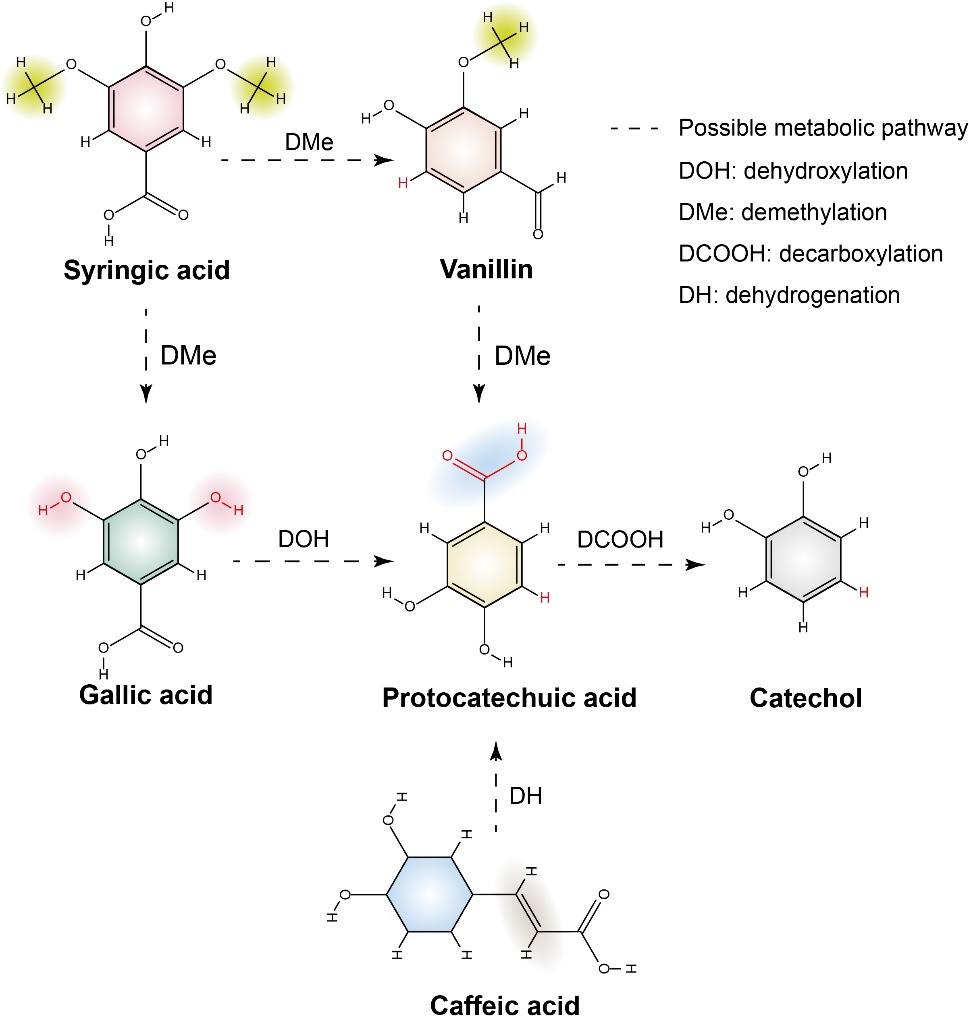


**Fig. S2.** The molecular docking results visualized by AutoDock 4.2.6 software: between 1-caffeoylquinic and α-amylase (A), vanillin and α-amylase (B), 1-caffeoylquinic and α-glucosidase (C), the enlargements were corresponding 3D visualization mechanism visualized by Discovery Studio 2016 software; (a), (b) and (c) were corresponding 2D visualization mechanism visualized by Discovery Studio 2016 software.


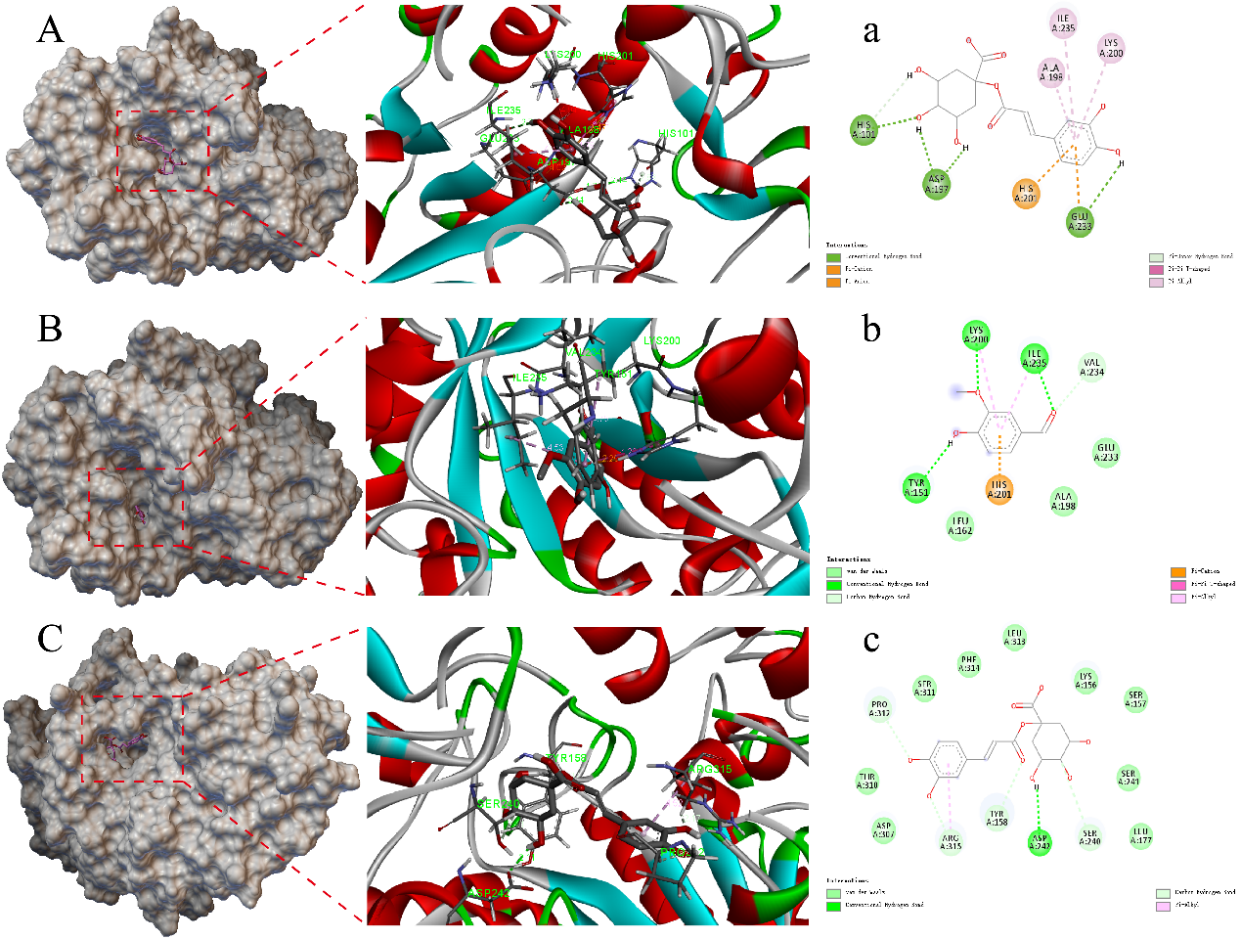


**Table S1**. Organic acids changes in red pitaya pulp fermented with various strains of *Lactobacillus* *^a^*

| Compounds (mg/mL) | Fresh fruits | p-Fresh fruits | *L. paracasei* LPC48 | *L. casei* LC122 | *L. plantarum* TWK 10 | *L. plantarum* LP-28 | *L. rhamnosus* LRH09 | *L. thermophilus* GRX02 | *L. acidophilus* LA1063 |
| --- | --- | --- | --- | --- | --- | --- | --- | --- | --- |
| Oxalic acid | 0.11±0.02f | 0.12±0.01c | 0.11±0.05f | 0.09±0.04 | 0.04±0.02f | 0.14±0.03de | 0.04±0.02g | 0.12±0.02g | 0.09±0.03g |
| Tartaric acid | 0.90±0.08b | 0.98±0.02g | 0.89±0.06b | 0.75±0.05b | 1.01±0.06b | 1.04±0.06b | 0.74±0.07b | 0.96±0.08c | 0.84±0.04d |
| Formic acid | 0.07±0.00g | 0.10±0.01bc | 0.11±0.01f | 0.17±0.03f | 0.18±0.01d | 0.07±0.00g | 0.13±0.01f | 0.48±0.02d | 0.03±0.00h |
| Ascorbic acid | 0.01±0.00h | 0.01±0.00a | 0.01±0.00h | 0.01±0.00h | 0.01±0.00g | 0.01±0.00h | 0.01±0.00h | 0.01±0.00h | 0.01±0.00i |
| Lactic acid | 0.41±0.01d | 0.36±0.02e | 6.24±0.11a | 5.41±0.08a | 5.75±0.03a | 4.72±0.04a | 6.72±0.03a | 3.57±0.05a | 4.95±0.03a |
| Acetic acid | 0.18±0.02e | 0.29±0.01d | 0.49±0.01d | 0.36±0.02d | 0.34±0.02c | 0.28±0.01c | 0.38±0.01d | 0.31±0.01e | 0.41±0.01e |
| Citric acid | 0.21±0.01e | 0.29±0.01d | 0.07±0.00g | 0.07±0.00g | 0.05±0.00f | 0.09±0.01f | 0.15±0.01f | 0.19±0.01f | 0.06±0.00g |
| Succinic acid | 0.64±0.02c | 0.76±0.01f | 0.69±0.03c | 0.45±0.02c | 0.35±0.01c | 1.15±0.08b | 0.62±0.03c | 0.99±0.06c | 1.36±0.08b |
| Malic acid | 1.69±0.05a | 1.58±0.02h | 0.21±0.01e | 0.23±0.01e | 0.20±0.02d | 0.16±0.01d | 0.22±0.01e | 1.22±0.02b | 0.98±0.03c |
| Pyruvic acid | 0.07±0.00g | 0.08±0.01b | 0.05±0.02g | 0.06±0.01g | 0.12±0.01e | 0.11±0.01e | 0.02±0.00g | 0.11±0.01g | 0.13±0.02f |
| Total | 4.29±0.18f | 4.57±0.06e | 8.87±0.23a | 7.60±0.10d | 8.05±0.12b | 7.77±0.05c | 9.03±0.13a | 7.90±0.18b | 8.86±0.21a |

*^a^* The row with different letters in a line represents a significant difference (p < 0.05).

**Table S2.** The utilization of free sugars in red pitaya pulp fermented with various strains of *Lactobacillus* *^a^*

| Samples | Fructose | | Glucose | | Maltose | | Total (mg/mL) |
| --- | --- | --- | --- | --- | --- | --- | --- |
|  | Content (mg/mL) | Utilization (%) | Content (mg/mL) | Utilization (%) | Content (mg/mL) | Utilization (%) |  |
| Fresh fruits | 17.14±0.21d | - | 42.22±0.41b | - | 13.05±0.26b | - | 72.41±2.12b |
| p-Fresh fruits | 17.38±0.19d | - | 43.67±0.39a | - | 13.98±0.22a | - | 75.05±1.84a |
| *L. paracasei* LPC48 | 15.98±0.18e | 8.06% | 27.17±0.23f | 37.77% | 12.70±0.18bc | 9.16% | 55.85±1.02f |
| *L. casei* LC122 | 12.73±0.18g | 26.75% | 33.79±0.33d | 22.62% | 11.62±0.12e | 16.88% | 58.14±1.21e |
| *L. plantarum* TWK 10 | 13.18±0.22f | 24.16% | 26.38±0.26g | 39.60% | 12.41±0.16c | 11.23% | 51.97±1.19g |
| *L. plantarum* LP-28 | 18.72±0.29c | -7.69% | 35.09±0.30c | 19.64% | 12.09±0.12d | 13.52% | 65.90±1.24cd |
| *L. rhamnosus* LRH09 | 11.79±0.31h | 32.14% | 27.31±0.24d | 37.47% | 12.05±0.14d | 13.81% | 51.15±1.41g |
| *L. thermophilus* GRX02 | 20.78±0.15a | -19.57% | 34.52±0.29cd | 20.96% | 12.04±0.14d | 13.88% | 67.34±1.28c |
| *L. acidophilus* LA1063 | 19.83±0.18b | -14.07% | 32.32±0.31e | 26.00% | 11.89±0.15de | 14.95% | 64.04±1.36d |

*^a^* The column with different letters in a line represents a significant difference (p < 0.05).

**Table S3**. Phenolics and flavonoids changes in red pitaya pulp fermented with various strains of *Lactobacillus* *^a^*

| Compounds (μg/mL) | Fresh fruits | p-Fresh fruits | *L. paracasei* LPC48 | *L. casei* LC122 | *L. plantarum* TWK 10 | *L. plantarum* LP-28 | *L. rhamnosus* LRH09 | *L. thermophilus* GRX02 | *L. acidophilus* LA1063 |
| --- | --- | --- | --- | --- | --- | --- | --- | --- | --- |
| Gallic acid | 1.48±0.05e | 1.45±0.03e | 5.28±0.13c | 5.55±0.11c | 3.31±0.08c | 3.23±0.05c | 5.19±0.14c | 3.34±0.06c | 4.16±0.08d |
| 2,4,6-Trihydroxybenzoic acid | 7.28±0.21a | 7.16±0.14a | 15.89±0.32a | 16.74±0.29a | 13.43±0.18a | 14.18±0.14a | 15.91±0.22a | 18.81±0.30a | 14.66±0.27a |
| 1-Caffeoylquinic acid | 0.48±0.02i | 0.35±0.01j | 2.56±0.12e | 1.54±0.08f | 2.63±0.15d | 2.80±0.14d | 2.45±0.11d | 2.62±0.12e | 2.61±0.10e |
| Protocatechuic acid | 0.76±0.01g | 0.69±0.02g | 1.10±0.03g | 1.07±0.02h | 1.01±0.03e | 1.04±0.02f | 1.04±0.02g | 1.19±0.04g | 0.98±0.03g |
| Catechol | 3.68±0.24b | 3.58±0.13b | 8.11±0.35b | 7.92±0.28b | 7.10±0.24b | 7.32±0.19b | 8.12±0.29b | 8.44±0.33b | 7.08±0.25c |
| Cyanidin-3-O-glucoside | 0.83±0.02f | 0.80±0.01f | 2.11±0.11f | 1.79±0.09e | 1.07±0.07e | 1.13±0.06e | 1.92±0.09e | 1.31±0.06f | 1.60±0.09 |
| Chlorogenic acid | 0.31±0.01k | 0.42±0.02i | 0.74±0.02i | 0.96±0.03i | 0.24±0.00h | 0.29±0.01i | 0.79±0.03h | 0.62±0.01i | 0.22±0.00i |
| Caffeic acid | 0.36±0.02j | 0.42±0.01i | 0.59±0.03j | 1.31±0.08g | 0.49±0.01g | 0.42±0.02f | 1.19±0.06f | 0.21±0.00j | 0.94±0.05g |
| Syringic acid | 0.53±0.01h | 0.50±0.01h | 0.85±0.02h | 0.93±0.04i | 0.81±0.02f | 0.82±0.02g | 0.69±0.01i | 1.00±0.01h | 0.69±0.01h |
| Vanillin | 1.99±0.05d | 1.78±0.03d | 2.54±0.08e | 2.55±0.07d | 3.35±0.09c | 3.34±0.08c | 2.54±0.04d | 3.18±0.07d | 1.92±0.04f |
| Quercetin-3-O-glucoside | 3.01±0.08c | 2.96±0.06c | 4.53±0.10d | 2.68±0.06d | nd | nd | 3.46±0.06c | 1.24±0.03f | 8.19±0.12b |
| Total | 20.71±0.08g | 20.11±0.09h | 44.30±0.13a | 43.04±0.08c | 33.44±0.10f | 34.57±0.09e | 43.30±0.09b | 41.96±0.11d | 43.05±0.13c |

*^a^* The row with different letters in a line represents a significant difference (p < 0.05).

nd = not detectable.

**Table S4.** Effect of fermentation on aroma compounds in red pitaya juice*^a^*

| Num | Name | Relative peak area (%) | | | | | | | | |
| --- | --- | --- | --- | --- | --- | --- | --- | --- | --- | --- |
|  |  | Fresh fruits | p-Fresh fruits | L. casei LC122 | L. paracasei LPC48 | L. rhamnosus LRH09 | L. acidophilus LA1063 | L. thermophilus GRX02 | L. plantarum LP-28 | L. plantarum TWK 10 |
| **Phenol** |  |  |  |  |  |  |  |  |  |  |
| 1 | 2,4-Di-tert-butylphenol | 33.42% | 18.02% | 32.78% | 56.47% | 51.26% | 62.38% | 56.12% | 57.72% | 65.98% |
|  | **Sum** | 33.42% | 18.02% | 32.78% | 56.47% | 51.26% | 62.38% | 56.12% | 57.72% | 65.98% |
| **Alcohols** |  |  |  |  |  |  |  |  |  |  |
| 1 | 1,14-Tetradecanediol | 0.23% | 0.00% | 0.00% | 0.00% | 0.00% | 0.00% | 0.76% | 0.00% | 0.00% |
| 2 | 2-Hexen-1-ol | 0.27% | 0.00% | 0.00% | 0.00% | 0.00% | 0.00% | 0.00% | 0.00% | 0.00% |
| 3 | Cyclobutanol | 0.00% | 0.00% | 0.00% | 0.00% | 0.00% | 0.00% | 0.00% | 0.00% | 0.81% |
| 4 | 4-Tetradecanol | 0.00% | 0.00% | 0.00% | 0.00% | 0.00% | 0.00% | 0.00% | 0.36% | 0.37% |
| 5 | 1,6-Heptadien-4-ol | 0.00% | 0.00% | 0.00% | 0.00% | 0.00% | 0.00% | 0.00% | 0.43% | 0.00% |
| 6 | 1-Nonanol | 0.00% | 0.00% | 0.00% | 0.00% | 1.36% | 0.00% | 0.00% | 1.24% | 0.00% |
| 7 | (S)-(+)-5-Methyl-1-heptanol | 0.00% | 0.00% | 0.00% | 0.00% | 0.00% | 0.00% | 0.00% | 0.39% | 0.00% |
| 8 | 1-Pentanol | 0.00% | 0.00% | 0.00% | 0.00% | 0.00% | 0.00% | 0.36% | 0.00% | 0.00% |
| 9 | 2,7-Octadiene-1,6-diol | 0.00% | 0.47% | 0.00% | 0.00% | 0.00% | 0.00% | 0.00% | 0.00% | 0.00% |
| 10 | Benzenemethanol | 0.00% | 0.00% | 1.21% | 0.00% | 0.00% | 0.00% | 0.00% | 0.00% | 0.00% |
| 11 | 7-methanoazulen-6-ol | 0.00% | 0.00% | 0.00% | 1.04% | 0.00% | 0.00% | 0.00% | 0.00% | 0.00% |
| 12 | 2,3-Epoxyhexanol | 0.00% | 0.00% | 0.00% | 0.34% | 0.00% | 0.00% | 0.00% | 0.00% | 0.00% |
| 13 | Linalool | 0.00% | 0.00% | 0.00% | 0.00% | 1.13% | 0.00% | 0.00% | 0.00% | 0.00% |
| 14 | Trifluoroacetyl-lavandulol | 0.00% | 0.26% | 0.00% | 0.00% | 0.33% | 0.00% | 0.00% | 0.00% | 0.00% |
|  | **Sum** | 0.50% | 0.73% | 1.21% | 1.38% | 2.81% | 0.00% | 1.12% | 2.42% | 1.18% |
| **Aldehydes** |  |  |  |  |  |  |  |  |  |  |
| 1 | Hexanal | 0.18% | 0.63% | 0.00% | 0.00% | 0.00% | 0.00% | 0.00% | 0.00% | 0.00% |
| 2 | Decanal | 0.45% | 0.00% | 0.00% | 0.00% | 0.00% | 0.00% | 0.00% | 0.00% | 0.00% |
| 3 | Benzaldehyde | 0.86% | 0.27% | 9.78% | 9.95% | 10.65% | 12.37% | 11.35% | 13.67% | 12.59% |
| 4 | 4-Propylbenzaldehyde diethyl acetal | 0.00% | 0.00% | 0.00% | 0.00% | 0.00% | 0.00% | 0.00% | 0.00% | 0.79% |
| 5 | Pentanal | 0.00% | 0.00% | 0.18% | 0.00% | 0.65% | 1.10% | 0.00% | 0.00% | 0.00% |
| 6 | Nonanal | 0.00% | 0.00% | 0.57% | 0.00% | 0.00% | 0.00% | 0.00% | 0.00% | 0.00% |
| 7 | 2-(3-Methyl-but-1-ynyl)-cyclohexene-1-carboxaldehyde | 0.00% | 0.00% | 0.00% | 0.37% | 0.00% | 0.00% | 0.00% | 0.00% | 0.00% |
|  | **Sum** | 1.49% | 0.89% | 10.53% | 10.32% | 11.30% | 13.48% | 11.35% | 13.67% | 13.38% |
| **Ketones** |  |  |  |  |  |  |  |  |  |  |
| 1 | 3,4-Hexanedione | 0.00% | 0.00% | 0.00% | 0.00% | 0.84% | 0.00% | 0.00% | 0.00% | 0.00% |
| 2 | 2'-Methyl-4'-propoxypropiophenone | 0.42% | 0.00% | 0.00% | 0.00% | 0.00% | 0.00% | 0.00% | 0.00% | 0.00% |
| 3 | 3,7-Octadien-2-one | 0.00% | 0.00% | 0.00% | 0.00% | 0.00% | 0.00% | 0.00% | 0.34% | 0.00% |
| 4 | 2(3H)-Furanone | 0.00% | 0.00% | 1.50% | 1.62% | 2.21% | 1.51% | 2.16% | 0.00% | 0.00% |
| 5 | p-Pentylacetophenone | 0.00% | 0.00% | 0.88% | 0.00% | 0.00% | 1.09% | 0.00% | 0.00% | 0.00% |
| 6 | 2-Butanone | 0.00% | 0.22% | 0.00% | 0.00% | 2.47% | 0.00% | 0.00% | 0.00% | 0.00% |
| 7 | 2,5-Cyclohexadiene-1,4-dione | 0.00% | 0.00% | 0.75% | 0.00% | 0.00% | 0.00% | 0.00% | 0.00% | 0.00% |
| 8 | Acetoin | 0.00% | 0.00% | 0.00% | 0.00% | 0.44% | 0.00% | 0.00% | 0.00% | 0.00% |
| 9 | Cyclopentadecanone | 0.00% | 1.65% | 0.00% | 0.00% | 0.00% | 0.00% | 0.00% | 0.00% | 0.00% |
| 10 | 1,3-Hexanedione | 0.00% | 0.14% | 0.00% | 0.00% | 0.00% | 0.00% | 0.00% | 0.00% | 0.00% |
| 11 | 2(3H)-Furanone, dihydro-5-propyl- | 0.00% | 0.00% | 0.00% | 0.00% | 0.00% | 0.00% | 0.00% | 0.00% | 2.11% |
| 12 | 5,9-Undecadien-2-one | 0.00% | 0.00% | 0.00% | 0.53% | 0.00% | 0.85% | 0.00% | 0.00% | 1.06% |
| 13 | 2-Methyl-1-(2-methyl-4-propoxyphenyl)propan-1-one | 0.00% | 0.00% | 0.00% | 0.00% | 0.00% | 0.00% | 0.00% | 0.70% | 0.00% |
| 14 | Oxacyclopentadecan-2-one | 0.00% | 3.10% | 0.00% | 0.00% | 0.00% | 0.00% | 0.00% | 0.00% | 0.00% |
| 15 | 4-(4-Methylbenzoylmethyl)-2H-1,4-benzoxazin-3(4H)-one | 0.00% | 0.29% | 0.59% | 0.00% | 0.00% | 0.00% | 0.00% | 0.00% | 0.00% |
|  | **Sum** | 0.42% | 5.41% | 3.71% | 2.15% | 5.95% | 3.45% | 2.16% | 1.05% | 3.17% |
| **Hydrocarbon** |  |  |  |  |  |  |  |  |  |  |
| 1 | Cyclopropane | 0.60% | 0.00% | 0.00% | 0.00% | 0.00% | 0.00% | 0.87% | 0.00% | 0.00% |
| 2 | Pentane | 0.71% | 0.46% | 1.84% | 0.00% | 2.14% | 0.39% | 0.67% | 0.00% | 0.00% |
| 3 | Limonene | 11.00% | 21.82% | 31.01% | 8.19% | 10.03% | 4.32% | 8.47% | 0.00% | 0.00% |
| 4 | Benzene | 2.01% | 1.31% | 0.23% | 1.42% | 1.31% | 0.00% | 1.66% | 1.71% | 1.95% |
| 5 | Tridecane | 5.04% | 8.52% | 0.00% | 0.00% | 0.00% | 0.00% | 0.00% | 0.00% | 0.00% |
| 6 | Longifolene | 4.12% | 5.73% | 0.00% | 0.00% | 0.00% | 0.00% | 0.00% | 0.00% | 0.00% |
| 7 | Hexane | 0.30% | 0.57% | 0.23% | 1.10% | 0.00% | 1.39% | 2.02% | 0.00% | 0.00% |
| 8 | 2-Hexene | 1.25% | 1.01% | 0.00% | 0.00% | 0.00% | 0.00% | 0.00% | 2.43% | 0.00% |
| 9 | 1,3-Dioxolane | 0.00% | 0.00% | 0.00% | 0.38% | 0.00% | 0.00% | 0.00% | 0.00% | 0.00% |
| 10 | Heptane | 0.25% | 0.00% | 0.00% | 0.30% | 0.00% | 0.00% | 0.00% | 0.00% | 0.00% |
| 11 | 1-Octadecyne | 2.17% | 0.00% | 0.00% | 0.00% | 0.00% | 0.00% | 0.00% | 0.00% | 0.00% |
| 12 | Cyclohexane | 1.52% | 0.71% | 0.00% | 0.00% | 0.00% | 0.00% | 0.00% | 0.00% | 0.00% |
| 13 | Cyclobutane | 0.00% | 0.00% | 0.00% | 0.00% | 0.00% | 0.00% | 0.00% | 0.00% | 0.89% |
| 14 | Decane | 0.00% | 0.00% | 0.00% | 0.00% | 0.00% | 0.00% | 0.00% | 1.55% | 1.17% |
| 15 | Octane | 0.00% | 0.00% | 0.00% | 0.52% | 1.36% | 0.43% | 0.44% | 0.37% | 0.00% |
| 16 | 1,3,6,10-Dodecatetraene | 0.00% | 0.00% | 0.00% | 0.00% | 0.00% | 0.00% | 0.00% | 0.54% | 0.00% |
| 17 | Propane | 0.00% | 0.00% | 0.00% | 0.00% | 0.00% | 0.99% | 1.06% | 1.00% | 0.00% |
| 18 | Cyclohexene | 0.00% | 0.55% | 0.00% | 0.00% | 0.00% | 0.00% | 0.00% | 0.88% | 0.00% |
| 19 | Cyclopentane | 0.00% | 0.00% | 1.48% | 0.00% | 0.35% | 0.00% | 1.25% | 0.00% | 0.00% |
| 20 | 1,3-di-n-Propyladamantane | 0.00% | 0.00% | 0.00% | 0.97% | 0.00% | 0.00% | 0.65% | 0.00% | 0.00% |
| 21 | 4,8-Dioxatricyclo[5.1.0.0(3,5)]octane | 0.00% | 0.00% | 0.76% | 0.00% | 0.00% | 0.71% | 0.33% | 0.00% | 0.00% |
| 22 | Ethylamine | 0.00% | 0.00% | 0.00% | 0.00% | 0.00% | 0.00% | 0.78% | 0.00% | 0.00% |
| 23 | 2,4-Dimethyl-1,5-diazabicyclo[3.1.0]hexane (cis) | 0.00% | 0.00% | 0.00% | 0.00% | 0.00% | 0.00% | 0.38% | 0.00% | 0.00% |
| 24 | Dibenz[b,f]][1,4]oxazepine | 0.00% | 0.00% | 0.00% | 0.00% | 0.00% | 0.00% | 0.35% | 0.00% | 0.00% |
| 25 | 1-Propene | 0.00% | 0.00% | 0.87% | 0.81% | 0.00% | 0.91% | 0.00% | 0.00% | 0.00% |
| 26 | 1,1-Hexylenedioxybutane | 0.00% | 0.00% | 0.00% | 0.00% | 0.00% | 0.34% | 0.00% | 0.00% | 0.00% |
| 27 | 2-Pentene | 0.00% | 0.39% | 0.00% | 0.00% | 0.96% | 0.00% | 0.00% | 0.00% | 0.00% |
| 28 | Toluene | 0.00% | 0.00% | 0.26% | 0.00% | 0.00% | 0.00% | 0.00% | 0.00% | 0.00% |
| 29 | Undecane | 0.00% | 0.00% | 1.09% | 0.00% | 0.00% | 0.00% | 0.00% | 0.00% | 0.00% |
| 30 | Bicyclo[3.1.1]heptane | 0.00% | 0.00% | 0.59% | 0.00% | 0.00% | 0.00% | 0.00% | 0.00% | 0.00% |
| 31 | Pentadecane | 0.00% | 0.00% | 0.38% | 0.00% | 0.00% | 0.00% | 0.00% | 0.00% | 0.00% |
| 32 | 1,1'-Biphenyl | 0.00% | 0.00% | 0.26% | 0.00% | 0.00% | 0.00% | 0.00% | 0.00% | 0.00% |
| 33 | Butane | 0.00% | 0.00% | 0.00% | 0.48% | 0.00% | 0.00% | 0.00% | 0.00% | 0.00% |
| 34 | Muurolene | 0.00% | 0.16% | 0.00% | 0.00% | 0.00% | 0.00% | 0.00% | 0.00% | 0.00% |
| 35 | Nonane | 0.00% | 0.28% | 0.00% | 0.00% | 0.00% | 0.00% | 0.00% | 0.00% | 0.00% |
| 36 | 1-Pentadecyne | 0.00% | 1.86% | 0.00% | 0.00% | 0.00% | 0.00% | 0.00% | 0.00% | 0.00% |
| 37 | Tricyclo[5.4.0.0(2,8)]undec-9-ene | 0.00% | 1.20% | 0.00% | 0.00% | 0.00% | 0.00% | 0.00% | 0.00% | 0.00% |
| 38 | 9,9-Dimethyl-9-silafluorene | 0.00% | 0.16% | 0.00% | 0.00% | 0.27% | 0.00% | 0.00% | 0.00% | 0.00% |
| 39 | Bicyclo[3.1.0]hex-2-ene | 0.00% | 0.00% | 0.00% | 0.00% | 0.00% | 0.00% | 0.00% | 2.51% | 0.00% |
| 40 | (1R,5R)-2-Methyl-5-((R)-6-methylhept-5-en-2-yl)bicyclo[3.1.0]hex-2-ene | 0.60% | 0.24% | 0.00% | 0.58% | 0.00% | 0.00% | 0.00% | 0.00% | 0.00% |
| 41 | Spiro[4.4]nona-1,3-diene | 0.98% | 0.00% | 0.00% | 0.00% | 0.00% | 0.00% | 0.00% | 0.00% | 0.00% |
|  | **Sum** | 30.54% | 44.96% | 39.00% | 14.75% | 16.43% | 9.49% | 18.93% | 11.00% | 4.00% |
| **Acids** |  |  |  |  |  |  |  |  |  |  |
| 1 | Sulfurous acid | 0.00% | 0.00% | 0.55% | 0.00% | 0.00% | 0.00% | 0.00% | 0.00% | 0.00% |
| 2 | Myristoleic acid | 2.15% | 0.00% | 0.00% | 0.00% | 0.00% | 0.00% | 0.00% | 0.00% | 0.00% |
| 3 | Palmitoleic acid | 9.93% | 24.53% | 0.00% | 0.00% | 0.00% | 0.00% | 0.00% | 0.00% | 0.00% |
| 4 | 1,2-Benzenedicarboxylic acid | 11.96% | 2.89% | 7.56% | 9.93% | 5.08% | 5.64% | 6.10% | 8.99% | 7.94% |
| 5 | Phthalic acid | 0.75% | 0.40% | 0.25% | 1.41% | 0.46% | 0.45% | 0.73% | 0.72% | 0.99% |
| 6 | Carbamic acid | 0.71% | 0.13% | 0.00% | 0.00% | 0.00% | 0.00% | 0.00% | 0.00% | 0.00% |
| 7 | Cyclopropanecarboxylic acid | 0.20% | 0.00% | 0.00% | 0.00% | 0.00% | 0.00% | 0.00% | 0.00% | 0.00% |
| 8 | Hexadecenoic acid | 6.12% | 0.00% | 0.00% | 0.00% | 0.00% | 0.00% | 0.00% | 0.00% | 0.00% |
| 9 | Benzoic acid | 0.00% | 0.52% | 2.04% | 0.00% | 0.00% | 3.62% | 0.00% | 0.00% | 1.02% |
| 10 | Oxalic acid | 0.00% | 0.38% | 0.00% | 0.00% | 0.00% | 0.00% | 0.00% | 0.00% | 0.48% |
| 11 | Propanoic acid | 0.00% | 0.00% | 0.17% | 0.00% | 0.00% | 0.00% | 0.31% | 0.32% | 0.45% |
| 12 | Butanoic acid | 0.00% | 0.00% | 0.44% | 0.46% | 0.00% | 0.50% | 0.00% | 0.77% | 0.00% |
| 13 | Acetohydroxamic acid | 0.00% | 0.00% | 0.00% | 0.00% | 0.00% | 0.00% | 0.00% | 0.56% | 0.00% |
| 14 | Dithiocarbonic acid | 0.00% | 0.19% | 0.00% | 0.00% | 0.00% | 0.00% | 0.00% | 0.00% | 0.00% |
| 15 | 2-Furancarboxylic acid | 0.00% | 0.00% | 0.00% | 0.00% | 0.35% | 0.00% | 0.00% | 0.00% | 0.00% |
| 16 | Isopropoxycarbamic acid | 0.00% | 0.00% | 0.00% | 0.00% | 0.00% | 0.33% | 0.00% | 0.00% | 0.00% |
| 17 | 4-Ethylbenzoic acid | 0.00% | 0.00% | 0.23% | 0.00% | 0.00% | 0.00% | 0.00% | 0.00% | 0.00% |
| 18 | Valeric acid | 0.00% | 0.00% | 0.00% | 0.54% | 0.00% | 0.00% | 0.00% | 0.00% | 0.00% |
| 19 | 3-Methylbenzoic acid | 0.00% | 0.00% | 0.00% | 0.00% | 0.42% | 0.00% | 0.00% | 0.00% | 0.00% |
| 20 | Carbonic acid | 0.00% | 0.00% | 0.00% | 0.00% | 0.57% | 0.00% | 0.00% | 0.00% | 0.00% |
| 21 | Phosphonofluoridic acid | 0.00% | 0.18% | 0.00% | 0.00% | 0.00% | 0.00% | 0.00% | 0.00% | 0.00% |
|  | **Sum** | 31.83% | 29.21% | 11.23% | 12.34% | 6.87% | 10.54% | 7.14% | 11.36% | 10.88% |
| **Esters** |  |  |  |  |  |  |  |  |  |  |
| 1 | 3-Methylbutan-2-yl 2,3,4,5,6-pentafluorobenzoate | 0.00% | 0.00% | 0.00% | 0.00% | 0.00% | 0.00% | 0.00% | 0.00% | 0.55% |
| 2 | Methyl 2-hydroxy-4-methylbenzoate | 0.00% | 0.00% | 0.00% | 1.20% | 2.27% | 0.00% | 1.52% | 1.69% | 0.00% |
| 3 | (E)-Hex-3-enyl isobutyl carbonate | 0.00% | 0.00% | 0.00% | 0.00% | 0.00% | 0.00% | 0.34% | 0.00% | 0.00% |
| 4 | n-Propyl acrylate | 0.00% | 0.00% | 0.22% | 0.00% | 0.00% | 0.00% | 0.00% | 0.00% | 0.00% |
| 5 | n-Hexyl acrylate | 0.00% | 0.00% | 0.00% | 0.31% | 1.55% | 0.00% | 0.00% | 0.00% | 0.00% |
| 6 | 8-Methyloctahydrocoumarin | 0.00% | 0.67% | 0.00% | 0.00% | 0.00% | 0.00% | 0.00% | 0.00% | 0.00% |
| 7 | N-Ethyl-2-amino-octadec-4-en-1,3-diol methaneboronate | 0.31% | 0.00% | 0.00% | 0.00% | 0.00% | 0.00% | 0.00% | 0.00% | 0.00% |
|  | **Sum** | 0.31% | 0.67% | 0.22% | 1.51% | 3.82% | 0.00% | 1.86% | 1.69% | 0.55% |
| **Ether** |  |  |  |  |  |  |  |  |  |  |
| 1 | Furan | 0.00% | 0.00% | 0.00% | 0.00% | 0.43% | 0.00% | 0.00% | 0.00% | 0.00% |
|  | **Sum** | 0.00% | 0.00% | 0.00% | 0.00% | 0.43% | 0.00% | 0.00% | 0.00% | 0.00% |

*^a^* Aroma compounds were tentatively identified by comparing their retention indices and mass spectrum with NIST11 library, and the retention indices (RI) were calculated according to the retention time of an n-alkane (C6-C24) obtained using same conditions.

The experiments were conducted in triplicate and the data in Table 5 were express as mean.

**Table S5**. The detailed docking parameters of α-amylase and α-glucosidase.

| Enzymes | Phenolics | Binding energy (Kcal/mol) | Number of binding to residues | Conventional hydrogen bond | Carbon hydrogen bond | Pi-Charge | | Pi-Alkyl | Pi-Pi T-shaped | Van der Waals |
| --- | --- | --- | --- | --- | --- | --- | --- | --- | --- | --- |
|  |  |  |  |  |  | Pi-Anion | Pi-Cation |  |  |  |
| α-Amylase | 1-Caffeoylquinic acid | -4.35 | 7 | HIS101, ASP197, GLU233 | - | HIS201, GLU233 | | ALA198, ILE235, LYS200 | HIS201 | - |
|  | Vanillin | -5.06 | 8 | TYR151, LYS200, ILE235 | VAL234 | HIS201 | | LYS200, ILE235 | HIS201 | LEU162, ALA198, GLU233 |
| α-Glucosidase | 1-Caffeoylquinic acid | -4.47 | 14 | ASP242 | PRO312, ARG315, TYR158, SER240 | - | | ARG315 | - | THR310, ASP307, SER311, PHE314, LEU313, LYS156, SER157, SER241, LEU177 |
